# First-Principle Electronic Properties of Dilute-P GaN_1−x_P_x_ Alloy for Visible Light Emitters

**DOI:** 10.1038/srep24412

**Published:** 2016-04-14

**Authors:** Chee-Keong Tan, Damir Borovac, Wei Sun, Nelson Tansu

**Affiliations:** 1 Center for Photonics and Nanoelectronics, Department of Electrical and Computer Engineering, Lehigh University, Bethlehem, PA 18015, USA

## Abstract

A study on the electronic properties of the dilute-P GaN_1−x_P_x_ alloy using First-Principle Density Functional Theory (DFT) calculations is presented. Our results indicate a band gap energy coverage from 3.645 eV to 2.697 eV, with P-content varying from 0% to 12.5% respectively. In addition, through line fitting of calculated and experimental data, a bowing parameter of 9.5 ± 0.5 eV was obtained. The effective masses for electrons and holes are analyzed, as well as the split-off energy parameters where findings indicate minimal interband Auger recombination. The alloy also possesses the direct energy band gap property, indicating its strong potential as a candidate for future photonic device applications.

In recent years, III-Nitride materials such as InGaN and AlGaN alloys, have been of particular interest due to the desirable electronic and optoelectronic properties, especially the direct and large band gap in the Brillouin Zone. Various modern technological applications based on the III-Nitride materials are developing, including light emitting diodes (LEDs), lasers, thermoelectric and solar energy devices, all enabled by advances in engineering innovations and material epitaxial processes^1^,^2^,^3^,^4^,^5^,^6^,^7^,^8^,^9^,^10^,^11^. Notably, the emergence of the III-Nitride materials earmarks the global revolution of lighting technology, which has recently been recognized by the 2014 Nobel Prize in Physics awarded for the works on III-Nitride based blue LEDs^12^.

In comparison to the progress in the InGaN and AlGaN alloys, the research involving the common-anion nitride-based ternary alloy such as the dilute-P GaNP alloy is still at its early stage^13^,^14^,^15^,^16^,^17^,^18^,^19^,^20^,^21^,^22^. The crystal growth of dilute-P GaNP was first successfully carried out by Igarashi and co-workers with halide vapor phase epitaxy^13^. The pioneering growth of GaNP alloys led to the successful incorporation of P impurity into the GaN alloy by using molecular beam epitaxial (MBE) technique, which resulted in a significant redshift of the photoluminescence peak emission wavelength^14^. Building on the promising features of the dilute-P GaNP alloy, Yoshida and co-workers managed to perform the growth of dilute-P GaNP alloys through metalorganic chemical vapor deposition technique (MOCVD) and fabricated a single quantum well light emitting diode device with the dilute-P GaNP alloy as the medium for the active region^15^. Even though these works demonstrated the possibility of realizing the growth of this alloy, the development on the dilute-P GaNP alloys has not progressed due to the lack of understanding in the physics behind the alloys.

It is important to note that numerous studies have been carried out in the fields of common-anion non-nitride-based semiconductor alloys such as InGaAs(N) alloys^23^,^24^,^25^, dilute-N GaPN alloys^26^,^27^,^28^,^29^,^30^ and GaAsP alloys^31^,^32^,^33^,^34^. In particular, the mixed PN-based alloys led to the indirect-to-direct band gap transition which opens up the opportunity in developing new light emitting devices^26^. In addition, the incorporation of Phosphorus impurities in the GaAs-based alloys has resulted in improved performance in the telecommunication and infrared device technologies^31^. Moreover, the use of dilute-N (In)GaAsN-based semiconductor alloys led to the making of state-of-the-art and low threshold laser devices for telecommunication applications^23^. The advances shown in these alloys are based on significant studies in the experimental works, understanding of the device physics and the electronic properties of the alloys.

Up to present, the electronic properties of the dilute-P GaNP alloy have yet to be understood, since the literature on the dilute-P GaNP alloy is severely limited. Recently, there has been a growing interest in the common-anion nitride-based material class, especially for the case of dilute-As GaNAs alloy^6^,^21^,^35^,^36^,^37^,^38^,^39^,^40^, where the analyses suggested the alloy as a potential novel material for light emitting devices, mainly due to the suppression of interband Auger recombination^38^,^40^. Due to the similarity between the dilute-P GaNP alloys and the dilute-As GaNAs alloys, it is thus important to conduct the analysis on the electronic properties of dilute-P GaNP alloys and determine their role in suppressing the interband Auger recombination. Understanding the electronic properties of the dilute-P GaNP alloys will provide the first key insights required for future implementation of the alloys in the light emitting applications.

In this work we report the findings on the electronic properties of dilute-P GaNP by using First-Principle Density Functional Theory calculations. Varying percentage of phosphorus (P) impurity concentrations from 0% P-content up to 12.5% P-content are introduced in the GaN bulk material. The computational method of the DFT calculation for the dilute-P GaNP alloys is discussed in section 2. The DFT-calculated band structures and electronic properties of the dilute-P GaNP alloy will be presented in section 3. Lastly, interband Auger recombination process in the dilute-P GaNP alloy is discussed through analysis of the resonance energy condition, which will be presented in section 4.

## Computational Method

**Figure 1:**
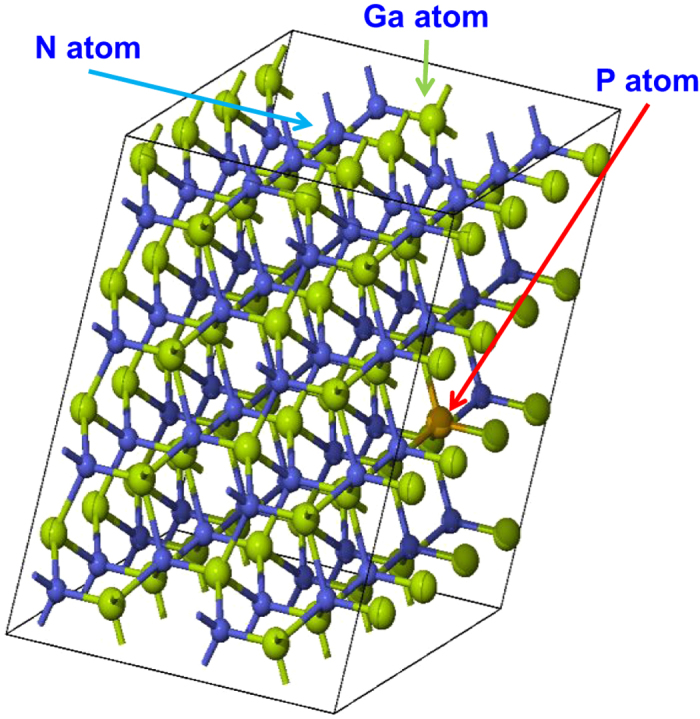
The 4 × 4 × 2 GaNP supercell consists of 64 Gallium (Ga) atoms, 64 Nitrogen (N) atoms and 1 Phosphorus (P) atom, corresponding to 1.56% P-content in the GaNP alloy.

For the DFT computations of the dilute-P GaNP alloys, supercell approach is applied. The supercell size was varied in our calculations to provide different P percentage for the GaNP alloy, which is similar to previous DFT work^38^. The supercell size ranges from 128 atoms to 32 atoms to form GaNP alloy with P-content from 1.56% to 12.5%. To illustrate the supercell approach, Fig. 1 shows a constructed GaNP alloy crystal structure, corresponding to a 4 × 4 × 2 supercell. The GaNP supercell consists of 128 atoms in total, comprised of 64 Ga atoms, 63 N atoms and 1 substituted P atom. The substitution of one P atom with an N atom in the 4 × 4 × 2 supercell corresponds to 1.56% P-content in the dilute-P GaNP alloy. For the band structure calculations, we employed the projector augmented wave (PAW) method as implemented in the MedeA-VASP software^41^. The local density approximation (LDA) is used to treat the exchange-correlation potential in the calculation. The electronic wave functions are described in plane wave basis with a cutoff energy of 400 eV and the structure is optimized by relaxing the atoms positions with the Hellman-Feynman force set to 0.02 eV/Å. External stress applied to the system was set to 0 GPa and the energy convergence tolerance was set to 1 × 10^−5^ eV/atom. The Monkhorst-Pack grid is gamma-centered and several high symmetry k-points were used in the band structure calculations. Different Monkhorst-Pack k-point meshes were generated in the calculations corresponding to different supercell sizes for the dilute-P GaNP alloys. The k-point meshes used in the calculations are 5 × 5 × 3 for 32-atom supercell, 4 × 4 × 3 for 72-atom supercell and 3 × 3 × 3 for 128-atom supercell, similar to that of previous DFT work for dilute-As GaNAs alloy^38^. The spin-orbit coupling was not considered in our calculations, as its effect in the wide band gap III-nitride semiconductor is insignificant. Note that the DFT calculation details have also been discussed elsewhere^38^.

### Band Structures and Band Parameters of Dilute-P GaNP Alloy

**Figure 2:**
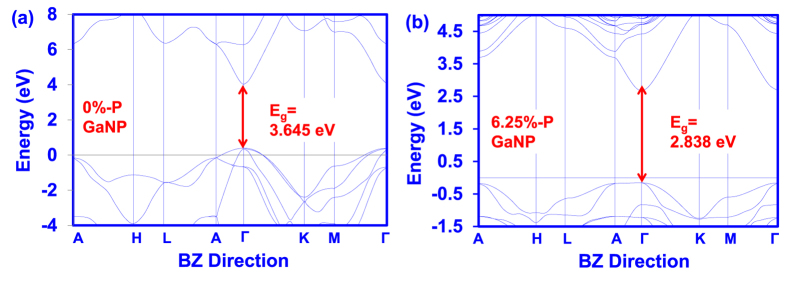
Band structures of GaNP alloy with (**a**) 0% and (**b**) 6.25% P-content. Energy band gap (E_g_) is the difference in energy between the conduction band minimum (CBM) and valence band maximum (VBM) at the gamma point in the Brillouin Zone.

Figure 2(a,b) show the DFT-calculated band structures of GaN and 6.25% P-content dilute-P GaN_0.9375_P_0.0625_ alloy respectively. As shown in Fig. 2(b), the conduction band minimum (CBM) and valence band maximum (VBM) for dilute-P GaNP are both located at the gamma (Γ) point in Brillouin Zone (BZ), indicating the direct band gap property of the alloys, an essential attribute for light emitting applications. Hence, the incorporation of P atoms in the GaN alloy does not lead to the transition from direct band gap to indirect band gap. This is different from the characteristics shown in dilute-N GaPN alloys where the incorporation of N atoms leads to the transition from indirect band gap to direct band gap^29^.

The energy band gap values of the dilute-P GaNP alloys were obtained by taking the value of the energy difference between the CBM and the VBM, as well as applying the scissor operator in order to correct the value originating from LDA calculations^42^. As presented in Fig. 2(b), the incorporation of 6.25% Phosphorus impurities into the GaN alloy led to an energy band gap reduction of ~0.81 eV compared to GaN. Such findings are analogous to the characteristics shown in dilute-As GaNAs alloys in which the incorporation of Arsenic impurities into the GaN alloy resulted in significant reduction of the band gap energy^38^.

**Figure 3:**
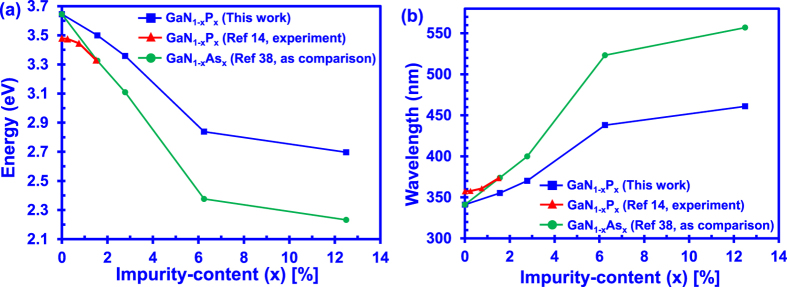
(**a**) Energy band gap and (**b**) Emission wavelength of dilute-P GaNP alloy from 0% up to 12.5% P-content. Experimental energy band gap of GaNP by Iwata *et al*.^14^ and DFT-calculated energy band gap of dilute-As GaNAs alloy^38^ are also plotted in the figure for comparison with the DFT-calculated energy band gap for dilute-P GaNP alloy.

The trend in reduction of the energy band gap with increasing P-content in the GaNP alloy is shown to be consistent at even higher P concentrations as shown in Fig. 3(a). At P-content of 12.5%, the band gap of dilute-P GaNP alloy is reduced by ~0.95 eV compared to GaN. The experimental data for the energy band gaps obtained by Iwata and co-workers were compared to our results for the dilute-P GaNP alloy^14^. In comparison, the calculated band gap energy data for dilute-As GaNAs alloy is also provided^38^. While the band gap reduction is less significant with the P impurity incorporation in the GaN, the trend of band gap energy reduction in GaNP alloy is similar to that of the dilute-As GaNAs alloy. There are discrepancies observed between the calculated and the measured results, even though the reducing trend of energy band gap is fairly similar. Such discrepancies potentially arise from the strain effect in the alloy and/or P-clustering effect in the experimentally grown GaNP alloy.

In addition, the band gap information of the dilute-P GaNP by Iwata and co-workers is obtained under different temperature conditions than ours. Our DFT calculation was performed for zero-temperature limit which is common in DFT calculations^43^,^44^. On the other hand, the band gap measurements by Iwata and co-workers were carried out at higher temperature of 77K. Note that the available experimental data on dilute-P GaNP is severely limited^14^ and the possibility for comparison at similar temperature is restricted at the current stage. Besides, the band gap problem is well known in DFT calculations. The band gap problem is attributed to the discontinuity in the exchange-correlation potential in the calculations, resulting in the underestimation of energy band gap in the material^45^. The underestimated energy band gap from DFT calculations can be corrected by using the scissor operator approach as implemented in this work^42^. Thus, these conditions would contribute to the discrepancies between the band gap results in comparison.

On the other hand, the band gap narrowing as a result of the incorporation of P impurities into the GaN alloy can be attributed mainly to the upward movement of the valence band of the alloy. It is important to note that the incorporation of Arsenic impurity in the GaN alloy has also been shown to be heavily affecting the valence band structure of the GaN alloy^38^. Similar effect is expected to be observed if the P impurity is incorporated into InGaN ternary alloy. Further study would be necessary to investigate the effect of the P atoms onto the InGaN alloy.

Figure 3(b) presents the emission wavelength as a function of P-content in the dilute-P GaNP alloy. As shown in Fig. 3(b), the light emission wavelength of the dilute-P GaNP alloy increases from 340 nm with 0%-P content to ~460 nm with 12.5%-P content in the dilute-P GaNP alloys. Specifically, the incorporation of 6.25%-P and 12.5%-P impurities into the GaN alloys lead to the 450 nm emission wavelength region. This suggests the possibility of the alloys as candidates for blue-light applications. The wavelength of the 12.5%-P GaNP alloy only constitutes a redshift of 20 nm as compared to the wavelength of the 6.25%-P GaNP alloy. The emission wavelength is expected to increase if more P impurities are incorporated into the dilute-P GaNP alloy. Note that the phase transformation phenomena has been observed and reported in the dilute-As GaNAs alloys when the As-content exceeds roughly 17%^37^. Since dilute-P GaNP alloys are similar to the dilute-As GaNAs alloys in that both P and As atoms are larger than the N atom, structural changes could possibly occur in the dilute-P GaNP alloys, when the percentage of Phosphorus impurities increases in the GaN alloy. Emission wavelength redshift in the dilute-P GaNP alloy could also be achieved by forming a III-Nitride active region heterostructure. Previous findings had shown that the conduction and valence bands will be bent due to the polarization field in the III-Nitride active region^4^,^35^,^36^. This leads to further reduction of the energy band gap, thus green light emission could possibly be achieved with low P-content in dilute-P GaNP active region.

**Figure 4:**
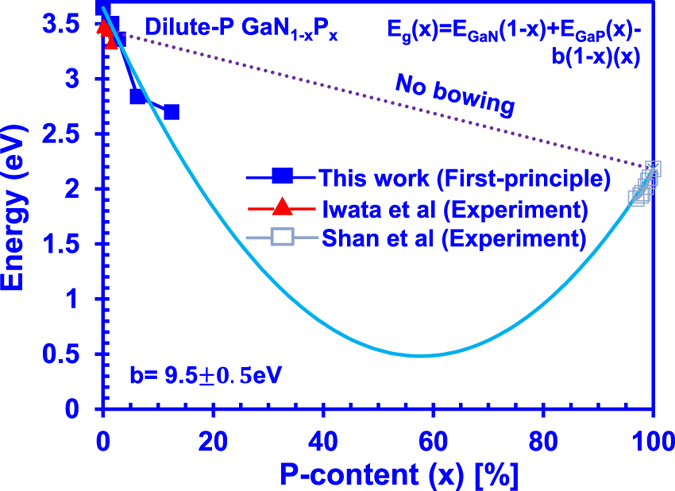
Comparison between our DFT calculations and experimental data, with corresponding bowing parameter of ~9.5 eV obtained through line fitting with the data.

Figure 4 presents the energy band gap of GaNP alloy in the full P-content composition range. The dotted line shown in Fig. 4 represents the energy band gap of GaNP alloys by taking into consideration the virtual crystal approximation model (VCA). On the other hand, the light blue solid line shown in Fig. 4 represents the band gap value of GaNP alloys while taking into account the bowing effect in the GaNP ternary alloys. As shown in Fig. 4, the VCA result deviates largely from the calculated and experimental energy band gap data, which shows that the bowing effect is significant in the GaNP alloys. By taking into the account the bowing parameter, the overall composition dependence of the alloy band gap is given as E_g_(x) = E_GaN_(1 − x) + E_GaP_(x) − b(1 − x)(x). The solid line in Fig. 4 shows a reasonable fitting with the experimental and the calculated energy band gap data for the GaNP alloys. Specifically, the band bowing parameter b is determined to be 9.5 ± 0.5 eV, which agrees well with the reported value of 9.31 eV by Iwata and co-workers^14^.

Along the quadratic curve provided by the composition dependent energy band gap equation in Fig. 4, the band gap energy of the GaNP alloys rapidly reduces from both ends, reaching a minimum of only 0.5 eV with 60% of P-impurity in the GaNP alloy. Note also that the formulated Hamiltonian matrices in the degenerate perturbation theory revealed that the composition dependency on the ternary alloy is more than just a simple quadratic function^35^, implying that the minimum energy band gap value in the GaNP ternary alloy requires further investigation. Nevertheless, this work is focused on the electronic properties of dilute-P GaNP alloy and its potential for optoelectronic device applications with a dilute limit of P-content. Further study would be necessary to investigate the effect of high-P-impurity content in the GaN alloy.

**Figure 5:**
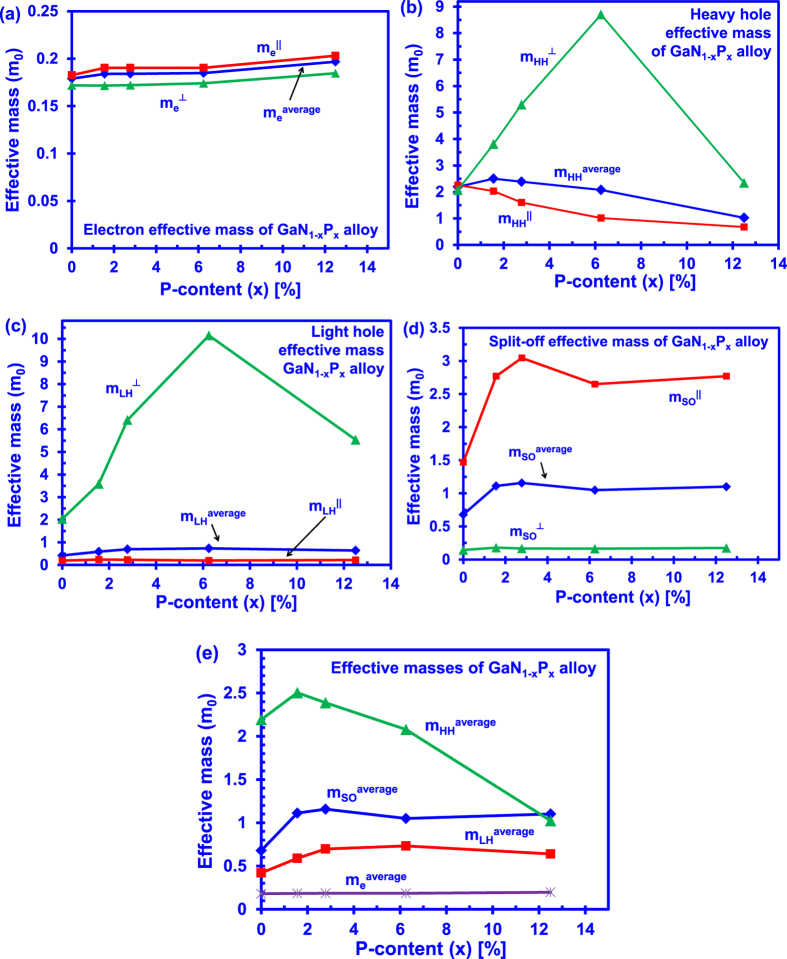
Carrier effective masses obtained through energy dispersion relation and parabolic line fitting with the calculated DFT band structures. (**a**) Electron (**b**) Heavy hole, (**c**) Light hole, (**d**) Split-off band and (**e**) Comparison of carrier effective masses, in which the heavy hole effective mass varies in a large order of magnitude compared to the electron effective mass.

Figure 5 presents the electron and hole effective masses of the dilute-P GaNP alloys from 0% up to 12.5% P-content. Effective mass parameters are obtained through the DFT-calculated band structures of the dilute-P GaNP alloys, in which the parabolic line fitting method is employed to fit the dispersion relation in k-space for the alloys^46^. For the GaN alloy, the electron effective mass is 0.2 m_0_ in the k_z_ direction and 0.17 m_0_ in the k_x_k_y_ direction. The effective mass for heavy hole, light hole and split-off have also been obtained via parabolic line fitting method, as shown in Fig. 5(b–d). Further details on the extraction of carrier effective mass from alloy band structures can be found elsewhere^46^.

As shown in Fig. 5(e), the average electron effective masses of the dilute-P GaNP alloys show an increasing trend from 0.17 m0 with 0% P-content to 0.18 m0 with 12.5% P-content, corresponding to a 6.67% effective mass increment. On the other hand, the average heavy hole effective masses of the dilute-P GaNP alloys show a general decreasing trend from 2.15 m_0_ with 0% P-content to 1 m0 with 12.5% P-content, leading to an effective mass reduction of 53.5%. The changes of the average electron effective masses are insignificant as compared to that of the average heavy hole effective mass for the dilute-P GaNP alloys, which is not the case in the dilute-nitride based (In)GaAsN and GaPN alloys^24^,^30^. It has been shown that there are significant changes occurring in the electron effective mass of the dilute-nitride based (In)GaAsN alloys where the hole bands remain unaffected as compared to the GaAs alloys^24^. The phenomena behind the significant electron effective mass changes is attributed to the addition of nitrogen impurities into the (In)GaAs alloys that leads to significant modification of the alloy conduction band, forming a strongly localized impurity energy band near the conduction band. In contrast to the properties of (In)GaAsN or GaPN alloys, when the Phosphorus atoms are incorporated into the GaN alloy, they form a localized impurity band with an energy level of 0.2 eV above the uppermost valence band^21^. Therefore, the hole bands in the dilute-P GaNP alloys are significantly modified, as the reconstruction of the valence bands in the alloys takes place due to the formation of localized P states.

**Figure 6:**
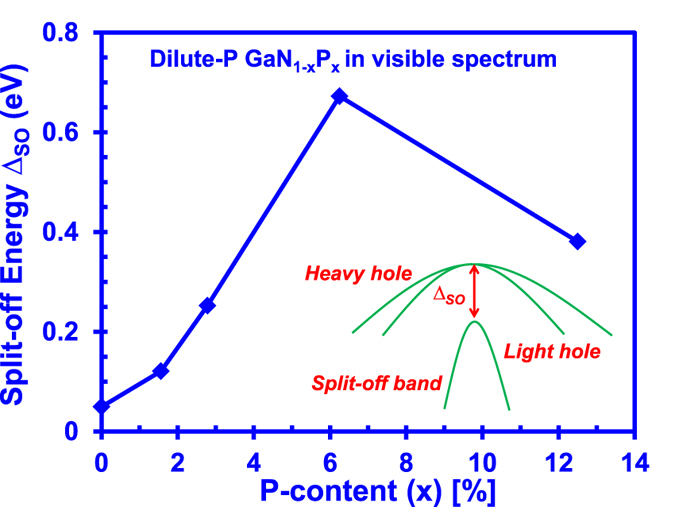
Split-off energy of dilute-P GaNP alloys from 0% up to 12.5% P-content, obtained through the energy difference between the valence band maximum (VBM) and the split-off band (SO) at gamma-point in Brillouin Zone.

Figure 6 presents the split off energy of the dilute-P GaNP alloys as a function of P-content. As shown in Fig. 6, the split-off energy of the dilute-P GaNP alloys has increased from 0.05 eV at 0%-P to 0.4 eV at 12.5%-P. The significant modification of ~0.35 eV in the split-off energy in the dilute-P GaNP alloys is quite significant as compared to the GaN and GaP alloys where the split-off energy value is only less than 0.1 eV^47^.

Based on our findings, the characteristics of the electronic properties shown in the dilute-P GaNP alloys are similar to that of the dilute-As GaNAs alloys^38^. From the MOCVD growth perspective, the GaNP alloy can be grown at temperature of about 850 °C–1050 °C^15^,^18^, which is compatible to the growth of GaN layer. The unique features provided by dilute-P GaNP alloys indicate the potential of the alloy as a candidate in the light emitting applications. It is important to note that there is still uncertainty on the electronic and optical properties of the dilute-P GaNP material at this early stage. Specifically, the incorporation of P atoms into the GaN is expected to result in additional defects, which will lower the radiative efficiency from this material system. Future experimental studies will be important to access the potential of the dilute-P GaNP material for the light emitting applications. Further investigations on the design of the GaNP-based heterostructure will be necessary in order to realize and optimize the performance of the GaNP-based devices.

### Interband Auger Recombination in the Dilute-P GaNP alloy

The findings on the energy band gap coverage by using dilute-P GaNP ternary alloys indicates possible realization of the alloys in the high power visible LED applications. Presently, the development of the InGaN LEDs is hindered by the efficiency droop issue in which the internal quantum efficiency of the devices reduces at high operating current density. The original cause of the efficiency droop issue in the InGaN LEDs is still inconclusive, despite extensive research in recent years that focused on carrier-related mechanisms, such as carrier leakage^48^,^49^,^50^ and Auger recombination^38^,^40^,^51^,^52^,^53^,^54^,^55^,^56^. Recent demonstrations of linear correlation between Auger electron and droop current in the InGaN LEDs by Iveland and co-workers indicate the significance of the Auger recombination process in the efficiency droop issue phenomena^52^. Recently, Delaney and co-workers suggested interband Auger recombination process as an important process in contributing to the efficiency droop issue in the green-emitting InGaN QWs^53^.

As the interband Auger recombination is pointed out as a possible key mechanism in leading to the efficiency droop issue in the InGaN QWs, the interband Auger process is primarily affected by the resonant energy, which is the energy difference between the energy band gap and the interband separation energy of the InGaN alloy. The interband Auger recombination process for the InGaN alloy is particularly significant when the resonant energy lies within the resonance condition (E_g_ − ∆ < 0.25 eV)^53^. Therefore, minimizing the interband Auger recombination process is important in order to ensure high efficiency in the nitride-based device.

Most recently, the dilute-As GaNAs alloy has been identified as a material system that suppresses the interband Auger recombination effect^38^,^40^. Note that the suppression of dominant interband Auger recombination in the InGaN alloy is restricted by the fundamental electronic band issue of the alloy and was unresolved prior to that of the application of dilute-As GaNAs material. The analytical Auger function model developed by Tan and co-workers indicated two key conditions which are (a) E_g_ > ∆ and (b) E_g_ < ∆; which govern the interband Auger recombination process^38^. Under condition (a), the Auger function is directly proportional to the exponential term of −(E_g_ − ∆)/2kT, whereas under condition (b), the Auger function is proportional to the exponential term of −(∆ − E_g_)/kT. In both expressions the parameter k corresponds to the Boltzmann constant while T corresponds to the room temperature (300 K). Further details in the working principle of the Auger function model have been discussed previously^38^. More importantly, the Auger function model demonstrated the capability of dilute-As GaNAs alloy in suppressing the interband Auger process, as the resonant energy of the alloy does not fulfill the resonance condition (E_g_ − ∆ < 0.25 eV). Due to the similarities in electronic properties of the dilute-P GaNP alloys and the dilute-As GaNAs alloys, it is important to determine the role of the interband Auger recombination in the alloys through the investigation on the related band properties of the dilute-P GaNP alloys.

**Figure 7:**
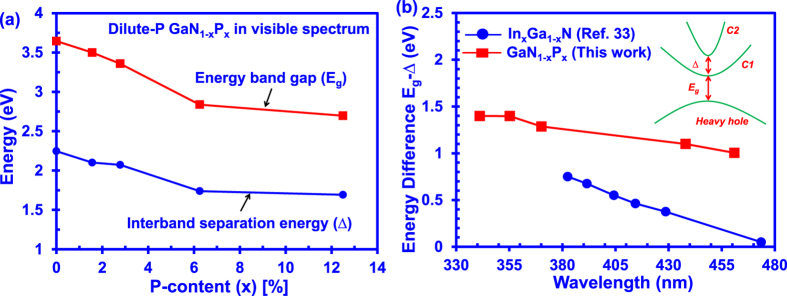
(**a**) Comparison between energy band gap E_g_ and interband separation energy ∆ of GaNP alloy from 0% up to 12.5% P-content, and (**b**) Comparison between the resonant energy E_g_ − ∆ of GaNP alloy and InGaN alloy as a function of emission wavelength.

Figure 7(a) shows the energy band gap and the interband separation energy of dilute-P GaNP and dilute-As GaNAs alloys from 0% up to 12.5% P and As-content respectively. As shown in Fig. 7(a), the interband separation energy of dilute-P GaNP alloys reduces from 2.25 eV at 0% P-content to 1.75 eV at 12.5% P-content, resulting in an energy reduction of 0.5 eV. Similarly, the energy band gap of the dilute-P GaNP alloys has a decreasing trend from 3.645 eV to 2.697 eV as the P-content increases in the alloy. In contrast, the interband separation energy of the InGaN alloy increases while the energy band gap reduces as the In-content increases in the alloy^53^.

The energy difference (E_g_ − ∆), or the resonant energy, as a function of emission wavelengths for the dilute-P GaNP alloys is shown in Fig. 7(b). As shown in Fig. 7(b), the resonant energy of the dilute-P GaNP alloys reduce slightly from 1.4 eV at wavelength of 340 nm to 1 eV at a wavelength of 460 nm. On the other hand, the resonant energy of the InGaN alloy reduces quicker over a shorter range of wavelengths, spanning from 0.8 eV at a wavelength of 380 nm to ~0 eV at wavelength of 480 nm^53^.

As presented in Fig. 7, the resonant energy (E_g_ − ∆) of all the dilute-P GaNP alloys from 0% to 12.5% P-content remain larger than 1 eV, which is far from satisfying the resonance energy condition (E_g_ − ∆ <0.25 eV). This corresponds to the off-resonance Auger process^38^, indicating minimal interband Auger recombination process in the dilute-P GaNP alloys. In addition, the P-content required in the GaNP alloys to achieve large band gap reduction is small in comparison to that of In-content in InGaN due to larger band gap bowing parameter in the GaNP alloys. Besides, the lattice mismatch between the GaNP alloy with 6.25%-P and the GaN alloy is ~1.2% which is comparable to that of InGaN alloy. Our findings on the dilute-P GaNP alloy show that the alloys present a new opportunity in the lighting technology due to their potential to serve as the active region material with suppressed interband Auger recombination in the visible spectral regimes.

## Conclusions

In summary, the band structures of the dilute-P GaNP alloys from 0%-P to 12.5%-P were calculated using First-Principle approach along with LDA approximation. The band structures of the GaNP alloys exhibit direct band gap property and the energy band gap of the alloys ranges from 3.645 eV to 2.697 eV with P-content varying from 0% to 12.5% respectively. The electronic band parameters for dilute-P GaNP alloys were also extracted, in which the band properties show similar characteristics as compared to the dilute-As GaNAs alloys, specifically related to the significant band gap narrowing effect attributed to the dilute amount of anion incorporation. The band structure parameters of dilute-P GaNP alloys are applicable for future device physics analysis or device technologies employing this alloy. The off-resonance condition analyzed in this work for the dilute-P GaNP alloy indicates minimal interband Auger recombination in the alloys, in contrast to the large interband Auger process reported for the conventional InGaN alloy^53^. This work presents a comprehensive understanding of the electronic properties of the dilute-P GaNP semiconductor, which provide a new direction in the pursuit of this alloy as the unconventional active material for visible photonic devices.

## Additional Information

**How to cite this article**: Tan, C.-K. *et al*. First-Principle Electronic Properties of Dilute-P GaN_1−x_P_x_ Alloy for Visible Light Emitters. *Sci. Rep.* **6**, 24412; doi: 10.1038/srep24412 (2016).
